# Antibiotic-Prescribing Patterns Among Patients With Respiratory Symptoms in the Eastern Province, Kingdom of Saudi Arabia

**DOI:** 10.7759/cureus.44298

**Published:** 2023-08-28

**Authors:** Nadira A Al-baghli, Ahmed Z Al Saif, Shorok A Al Dorazi, Mariam H Zainaldeen, AbdulMuhsen H Alameer, Slava Albaghli, Ahmad M Al-Dawood, Salma M Buhelaiga, Batool S Alsalim, Ali A Rabaan

**Affiliations:** 1 Public Health Administration, Dammam Health Network, Dammam, SAU; 2 Keep Well, Model of Care, Eastern Health Cluster, Dammam, SAU; 3 Directorate of Infection Prevention and Control, General Directorate of Health Affairs in Eastern Province, Dammam, SAU; 4 Dentistry, Dammam Health Network, Dammam, SAU; 5 Pathology and Laboratory Medicine, King Saud Medical City, Riyadh, SAU; 6 Family Medicine, Dammam Health Network, Dammam, SAU; 7 Molecular Microbiology, Johns Hopkins Aramco Healthcare, Dhahran, SAU

**Keywords:** saudi arabia, centor criteria, respiratory tract infection, prescribing, antibiotics

## Abstract

Background

Upper respiratory tract infections (URTIs) represent the most common diagnosis in ambulatory care settings. Some of these infections are properly treated with antibiotics, but evidence points to an inappropriate overuse of antibiotics in URTI management. This overuse is linked to antibiotic resistance, drug-related adverse effects, and increased costs.

Objective

This study evaluated the prevalence and predictors of antibiotic prescription for patients with URTI symptoms at the primary healthcare centers (PHCCs) and pediatric emergency department (ED) of the Maternity and Children Hospital (MCH) in Dammam, Saudi Arabia.

Methods

A prospective study was conducted in the PHCCs and pediatric ED of MCH. Trained physicians collected data on patients with URTI symptoms aged three years and older. Scores based on modified Centor criteria were calculated, and rapid antigen detection tests (RADTs) were conducted for all study participants.

Results

Out of 469 patients with a URTI, 141 (30.1%) received a prescription for an antibiotic, with a smaller proportion in the PHCCs (n=85; 24.4%) than in the pediatric ED (n=56; 46.3%). The main significant predictors of antibiotic prescription in terms of odds ratio (OR) and 95% confidence interval (95%CI) were a positive RADT result (OR=41.75, 95%CI=4.76-366.28), the presence of tonsillar exudate (OR=5.066, 95%CI=3.08-8.33), tender and/or swollen anterior cervical lymph nodes (OR=4.537, 95%CI=1.96-10.54), and fever (OR=3.519, 95%CI=2.33-5.31). A higher Centor score was also a predictor (2 to 5 vs. −1 to 1) (OR=2.72, 95%CI=1.8-4.12). The absence of a cough was not a significant predictor (OR=1.13, 95%CI=0.74-1.72).

Conclusions

Although a positive RADT increased the likelihood that a patient would be prescribed an antibiotic at the time of assessment, most antibiotic prescriptions were not justified. To control expenses, prevent adverse effects, and limit the spread of antibiotic resistance, efforts should be made to reduce unnecessarily high antibiotic usage.

## Introduction

The inappropriate use of antimicrobial agents has been linked to antibiotic resistance, which is a global public health concern [1]. Upper respiratory tract infections (URTIs) represent the most common diagnosis in outpatient visits and the most frequent reason for prescribing antibiotics, despite viruses being the most prevalent cause of URTIs [2]. Additionally, 19.3% of emergency department visits related to drug-related adverse effects are due to antibiotics, with many of these incidents involving an allergic response [3]. Furthermore, antibiotic misuse and its consequences increase healthcare costs and place a strain on healthcare systems [1].

The Kingdom of Saudi Arabia (KSA) has a high rate of antibiotic use, ranging from 41% to 92% of total use, and the misuse is especially prevalent among children [4], which was prior to the law promulgated in the KSA, by Ministry of Health and the Executive Regulations of Health Practice Law in April 2018, which prohibit the sale of antibiotics without a physician’s prescription [5]. This policy targets antimicrobial management and aims to lower the prevalence of self-medication and compels patients to seek advice from healthcare professionals. However, a study done by Al-Jedai et al. revealed that the new policy has had no long-term impact, possibly due to easy access to healthcare facilities in the KSA and the ability to obtain antibiotics for free at healthcare facilities if prescribed by physicians [6].

The aim of the current study was to discover the determinants of antibiotic prescription for URTIs in primary healthcare centers (PHCCs) and a pediatric emergency department (ED) affiliated with a hospital in the KSA. Most of the published literature regarding antibiotic prescription is retrospective or derived from records. Furthermore, these studies do not include Centor criteria or rapid antigen detection test (RADT) results. A prospective study to determine the factors influencing physicians’ practices in prescribing antibiotics for a disease mostly caused by viral infection could provide insight into the factors underlying the problem of antibiotic misuse. Therefore, the objective of this study was to explore the patterns and factors influencing antibiotic prescription for patients with URTIs.

This study is expected to increase awareness about the overuse of antibiotics among general practitioners and pediatric physicians covering EDs and PHCCs. The results of this study could be used in planning and assessing the need for training and education in the judicious use of antibiotics and the proper treatment of URTIs.

## Materials and methods

A sequential sample was collected prospectively from the ED of Maternity and Children Hospital (MCH), which is a secondary care hospital in Dammam, KSA, and from three PHCCs in the same city. Visitors aged three years and older with respiratory tract symptoms, such as sore throat, blocked or runny nose, cough, or any sign of pharyngitis were included.

Data collection tool

Participants or their guardians were asked by well-trained physicians about their willingness to participate in the study. Those willing to participate were interviewed by their physician, who used a questionnaire created for this study. The questionnaire was previously validated by four expert consultants in infectious disease, community medicine, public health, and family medicine. After the questionnaire was completed, a standard throat swab was collected using polyester (Dacron)-tipped swabs. A rapid test for group A streptococcus infection was conducted using Alere TestPack Plus Strep A with On Board Controls, which is a rapid immunoassay for the qualitative detection of the group A streptococcal (group A strep) antigen from throat swab specimens for the diagnosis of group A strep pharyngitis.

Modified Centor scores were computed based on the criteria presented in Table 1 [7].

**Table 1:** Modified Centor criteria and scores

Criteria	Score
Age (years)	
3–14	+1
15–44	0
≥45	−1
Swelling or exudates on tonsils	Yes (+1)
Tender and/or swollen anterior cervical lymph nodes	Yes (+1)
Temperature >38°C (100.4°F)	Yes (+1)
Cough present	No (+1)

Ethical considerations

The questionnaire and protocol were approved by the Dammam Medical Complex, KSA, the Institutional Review Board (Approval No.: H-05-D-107), and by the relevant authorities before conducting the study. The aim of the study was explained to the selected population. Prior to their participation in the study, consent was obtained from the patients or, in the case of children, from their parents or guardians. If children were able to communicate, their approval for participation was obtained. All names and data were handled with respect for confidentiality.

Data processing and analysis

Data for all variables were checked for accuracy and completeness and then coded. The data were then entered into a personal computer using Statistical Package for the Social Sciences (SPSS) version 26 (IBM Corp., Armonk, NY) for cleaning and analysis. The chi-square test or Fisher’s exact test was used for the bivariate analysis of categorical variables. Binary logistic regression was performed for multivariate analysis, where a p-value of <0.05 was considered statistically significant.

## Results

From March 13, 2022, to September 30, 2022, data were collected from 469 patients with respiratory symptoms at the PHCCs and ED of MCH. As shown in Table 2, the majority of the participants were Saudi (n=447; 95.5%), and about one-third (38.4%) were aged 3-14 years. A minority of patients were healthcare providers (8.0%). Eighty-nine participants (19%) had at least one chronic illness, and only 44 (9.4%) reported being a smoker.

**Table 2:** Characteristics of the study group by study location * Chi-squared or Fisher’s exact test. PHCC=primary healthcare center; ED=emergency department; RADT=rapid antigen detection test

Characteristics	Total n (%)	PHCCs, n=348 n (%)	ED, n=121 n (%)	p-value*
Sex (male)	220 (47.0)	165 (47.6)	55 (45.5)	0.691
Nationality (Saudi)	447 (95.5)	331 (95.4)	116 (95.9)	0.827
Age group				<0.001
3–14 years	180 (38.4)	71 (20.4)	109 (90.1)	
15–44 years	194 (41.4)	182 (52.3)	12 (9.9)	
>44	95 (20.3)	95 (27.3)	0 (0)	
Healthcare personnel	37 (8.0)	33 (9.7)	4 (3.3)	0.027
Has chronic diseases	89 (19.0)	77 (22.1)	12 (9.9)	0.003
Current smoker	44 (9.4)	44 (12.7)	0 (0)	<0.001
History of receiving treatment with antibiotics in the past 28 days	37 (7.9)	17 (4.9)	20 (16.5)	<0.001
Presenting symptoms				
Cervical lymph node	25 (5.3)	2 (8.0)	23 (92)	<0.001
Tonsillar exudate	84 (17.9)	2 (2.4)	82 (97.6)	<0.001
Fever	190 (40.5)	82 (43.2)	108 (56.8)	<0.001
Absence of cough	151 (32.2)	105 (69.5)	46 (30.5)	0.112
Positive RADT result	19 (4.1)	2 (0.6)	17 (14.0)	<0.001
Antibiotics prescribed in the current visit	141 (30.1)	85 (24.4)	56 (46.3)	<0.001

Regarding the Centor criteria, the most commonly reported symptoms were fever and cough in 190 (40.5%) and 318 (67.8%) participants, respectively. RADT was positive in a small proportion of participants (n=19; 4.1%).

Thirty-seven (7.9%) of the patients included in the study had received antibiotics within the previous 28 days, and 141 (30.1%) received antibiotics by the end of the current visit, comprising 85 (24.4%) from the PHCCs and 56 (46.3%) from the ED.

A comparison between the PHCCs and ED patients revealed a significant difference (p<0.05) across almost all relevant factors. ED patients were more likely to be younger and have more symptoms (both p<0.05). Moreover, ED patients were significantly more likely to have symptoms suggestive of strep A infection, positive RADT results, and a history of receiving antibiotics in the past 28 days and to be prescribed antibiotics by the current visit (all p<0.05). The PHCC patients were more likely to be healthcare providers, have chronic illnesses, and be smokers (all p<0.05).

Unadjusted bivariate analysis showed that younger patients, those with chronic illnesses, and those with positive RADT results were more likely to be prescribed antibiotics during the current visit (Table 3). Tender anterior cervical lymph nodes, tonsillar exudates, and fever, but not absence of cough, were also associated with a tendency for antibiotics being prescribed. In addition, healthcare providers had lower odds of receiving antibiotics compared with other groups.

**Table 3:** Multivariate analysis for predictors of antibiotics prescription *Significant. **Replaced with Centor Score. PHCC=primary healthcare center; ED=emergency department; RADT=rapid antigen detection test; OR=odds ratio; CI=confidence interval

Variable	Unadjusted OR (95%CI)	Adjusted OR (95%CI) Model 1	Adjusted OR (95%CI) Model 2	Adjusted OR (95%CI) Model 3
Place of interview (ED vs. PHCC)	2.67 (1.73–4.12)*	0.24 (0.07–0.76)*	1.25 (0.66–2.38)	1.35 (0.72–2.55)
Sex (male)	0.87 (0.59–1.3)	-	-	-
Nationality (Saudi)	0.56 (0.23–1.36)	-	-	-
Age group (years)			**	**
3–14	1 (Reference)	1 (Reference)		
15–44	0.47 (0.298–0.74)*	1.56 (0.78–3.11)		
>44	0.76 (0.45–1.29)	2.14 (0.88–5.2)		
Healthcare personnel	0.19 (0.06–0.62)*	0.25 (0.07–0.87)*	0.26 (0.08–0.87)*	-
Comorbidities	1.68 (1.04–2.71)*	1.6 (0.81–3.15)	1.93 (1.13–3.29)*	1.03 (0.42–2.52)
Smoking	1.23 (0.64–2.38)	-	-	-
History of receiving treatment with antibiotic in last 28 days	0.75 (0.34–1.63)	-	-	-
Positive RADT result	47.62 (6.29–333.33)*	41.75 (4.76–366.28)*	26.3 (3.4–203.62)*	30.8 (3.92–241.71)*
Suggestive symptoms:			**	**
Cervical lymph node	4.537 (1.96–10.54)*	3.42 (1.1–10.59)*		
Tonsillar exudate	5.066 (3.08–8.33)*	8.16 (2.68–24.79)*		
Fever	3.519 (2.33–5.31)*	3.37 (1.91–5.94)*		
Absence of cough	1.13 (0.74–1.72)	0.86 (0.52–1.43)		
Centor Score (2 to 5 vs. −1 to 1)	2.72 (1.8–4.12)*	-	2.02 (1.1–3.73)*	2.28 (1.23–4.21)*

In a primary multivariate model including setting, age group, chronic illnesses, RADT result, and symptoms, the significant predictors of receiving antibiotics were visiting the ED (OR=0.24, 95%CI=0.07-0.76), not being healthcare personnel (OR=0.25, 95%CI=0.07-0.87), having a positive RADT result (OR=41.75, 95%CI=4.76-366.28), and having suggestive symptoms (i.e., cervical lymph node, tonsillar exudate, and fever). Absence of cough was not associated with higher odds of receiving antibiotics in this model (OR=0.86, 95%CI=0.52-1.43), as seen in Table 3.

In a second multivariate model that replaced age groups and symptoms with the Centor score as a binary predictor (i.e., a score of 2 or more vs. 1 or less), a high Centor score of 2 or more doubled the odds of being prescribed antibiotics (OR=2.02, 95%CI=1.1-3.73). Except for setting (OR=1.25, 95%CI=0.66-2.38), all other variables, including being healthcare personnel (OR=0.26, 95%CI=0.08-0.87), having a chronic illness (OR=1.93, 95%CI=1.13-3.29), and the strongest predictor, having a positive RADT result (OR=26.3, 95%CI=3.4-203.62), were significant in this model.

A third model was run after restricting age to 3-14 years and excluding job category as a predictor. Model 3 confirmed the findings of model 2, as a positive RADT result and a Centor score of 2 or more were significantly associated with an antibiotic prescription, whereas setting and comorbidities were not.

## Discussion

The current study found that 30.1% of patients with URTI received antibiotics, which is relatively lower than percentages reported previously in the KSA [8-11] and most other studies worldwide such as 85% in Jordan [12], up to 80% in Egypt [13], 83.7% in China [14], 38.7% in the United Kingdom [15], 75.8% in Iceland, and 59.3% in Denmark [16]. The results of this study suggest a decreasing tendency to prescribe antibiotics over time. For example, antibiotic prescription for URTIs was reported to be 87.8% in a study by el-Gilany [10] in the northern region of Saudi Arabia in 1998, whereas this rate dropped to 79% in a study done in 2021 by Olwi and Olwi [11]. This decrease may be attributed to the regulations and local health practices made clear by the Ministry of Health, in addition to improvements in physicians’ understanding of regulations, communication to parents to raise their awareness about the risks associated with antibiotic misuse and to convince them to forego unnecessary antibiotic use, and restrictions on the sale of drugs without a prescription. Olwi and Olwi reported a reduction in the prescription of antibiotics, yet they also showed that only 28% of prescriptions could be justified by evidence [11]. In the current study, the unjustified use of antibiotics had an even higher rate than in the previously mentioned studies, with 74 (52.5%) participants who were prescribed antibiotics having a Centor score of less than 2, and 123 (87.2%) having a negative RADT result. These results reflect the fact that despite the reduction in antibiotic use, misuse still persists. Further, this study highlights that physicians have greater responsibility for the possible misuse, as over-the-counter purchases of antibiotics have been forbidden since April 2018.

The percentage of antibiotic prescriptions among visitors with URTI symptoms varies among healthcare facilities with different scopes of services, with lower rates in PHCCs (24%) than in the pediatric ED (46.3%). The intensity of symptoms and signs among ED visitors compared with PHCC visitors can explain the higher rate of antibiotic prescription in the ED, as supported by the results of this study. Statistically, setting was a significant factor when adjusted for age groups but not for Centor score, indicating that significance was related to symptoms and signs rather than setting. Furthermore, parents who bring their children to the ED are typically very worried about their health and may place greater pressure on physicians to prescribe antibiotics. In a 2018 KSA survey, the majority of physicians said that they feel pressured when patients ask for an antibiotic prescription [17]. Moreover, guardians might bring their child to the ED after the child’s symptoms do not improve or become worse. This possibility is apparent in the data presented in this study, given that 16.5% of ED visitors had received antibiotics in the past 28 days, which indicates that medical treatment was previously sought, as antibiotics have to be prescribed by a physician in the KSA. Although the rate of antibiotic prescription in the ED was more than that in the PHCCs, the former was still in line with most studies that examined antibiotic prescription in EDs [8,18]. However, the rate far exceeded the recommendation of the European Surveillance of Antimicrobial Consumption that the proportion of URTIs treated with antibiotics should be 20% or lower [19].

Other than being a visitor to the ED, having a chronic illness was the other predictor of receiving antibiotics, which might reflect a belief that this group is vulnerable to bacterial infections that must be bolstered by antibiotics. Another predictor of prescribing antibiotics was the presence of fever, although this symptom does not differentiate between bacterial and viral infections [7]. However, the majority of physicians indicate that a high fever is a sign that prompts them to prescribe antibiotics in the absence of laboratory confirmation [17]. 

Guidelines for the proper management of URTIs do not appear to be adequate to dramatically reduce the unnecessary prescription of antibiotics. This problem could be the result of the difficulty in the implementation of the guidelines in daily clinical practice because of other factors that enhance antibiotic prescription, including patient behavior and lack of information or understanding [20,21]. For example, parents might still insist on antibiotics for URTIs despite being well-informed about antibiotic misuse [22]. A systematic review of the factors leading to inappropriate prescription of antibiotics categorized them into the following groups: physician-related factors (e.g., inadequate knowledge, misconceptions about evidence-based prescribing, perceived patient expectations, diagnostic uncertainty, and confidence regarding adherence to appropriate prescribing behaviors), patient-related factors (e.g., signs and symptoms at the time of the prescription), and healthcare system/resource-related factors (e.g., time restrictions, patient load, cost savings, and influence of pharmaceutical companies) [23].

A systemic review of the effectiveness of physician-targeted interventions to improve appropriate antibiotic use for URTI revealed that, compared with written patient information and audit/feedback, educational meetings are more labor intensive but also appear to be more successful [24]. Furthermore, according to a meta-analysis on patient-oriented interventions that improve antibiotic-prescribing practices in URTIs, patients accept not receiving antibiotics as long as they are being treated adequately, examined, and given a thorough explanation [25]. A review of antimicrobial stewardship initiatives aiming for the responsible use of antimicrobial agents revealed that they improved clinical outcomes and lowered the frequency of drug-related adverse events and cost outcomes in most studies [26].

Multiple interventions to increase appropriate antibiotic prescription in the KSA (e.g., educational materials, feedback, and audit) have been associated with significant improvements [27]. By making the purchase of antimicrobial drugs without a prescription illegal, the KSA took a significant step toward reducing their misuse. However, when prescribing antibiotics in ambulatory care, doctors must support these national standards by following evidence-based practices. Furthermore, the effectiveness of such policies would also be increased by laws requiring the documentation of the indication for the prescribed antibiotics, as well as increased patient awareness.

Limitations

The main limitation of this study was the inability to mask the RADT results from the physicians, which may have affected their decision to prescribe antibiotics. In addition, it was not possible to implement a systematic random selection of patients given the context of a busy clinic.

## Conclusions

To the best of our knowledge, this study is the first prospective investigation combining Centor criteria and RADT results to assess the practice of antibiotic prescription. The positive impact of restricting over-the-counter sale of antimicrobial drugs could be strengthened by addressing the root causes of antibiotic misuse, promoting physicians’ rational use of antibiotics through education and training on best evidence-based practices, and auditing their performance in a way that does not interfere with patient care and individualized treatment. Furthermore, supporting physicians’ decision-making with improved availability of RADT assessment when Centor criteria require confirmation can help minimize the prescription of unnecessary antibiotics.

## References

[1] Harris AM, Hicks LA, Qaseem A (2016). Appropriate antibiotic use for acute respiratory tract infection in adults: advice for high-value care from the American College of Physicians and the Centers for Disease Control and Prevention. Ann Intern Med.

[2] Shehab N, Patel PR, Srinivasan A, Budnitz DS (2008). Emergency department visits for antibiotic-associated adverse events. Clin Infect Dis.

[3] Cohen AL, Budnitz DS, Weidenbach KN, Jernigan DB, Schroeder TJ, Shehab N, Pollock DA (2008). National surveillance of emergency department visits for outpatient adverse drug events in children and adolescents. J Pediatr.

[4] Alnemri A, Almaghrabi R, Alonazi N, Alfrayh A (2016). Misuse of antibiotic: a systemic review of Saudi published studies. Curr Pediatr Res.

[5] (2022). Ministry of Health, Saudi Arabia: MOH warns against selling antibiotics without prescription. https://www.moh.gov.sa/en/Ministry/MediaCenter/News/Pages/news-2018-04-17-004.aspx.

[6] Al-Jedai AH, Almogbel Y, Eljaaly K (2022). Restriction on antimicrobial dispensing without prescription on a national level: Impact on the overall antimicrobial utilization in the community pharmacies in Saudi Arabia. PLoS One.

[7] McIsaac WJ, White D, Tannenbaum D, Low DE (1998). A clinical score to reduce unnecessary antibiotic use in patients with sore throat. CMAJ.

[8] Alanazi MQ, Al-Jeraisy MI, Salam M (2015). Prevalence and predictors of antibiotic prescription errors in an emergency department, Central Saudi Arabia. Drug Healthc Patient Saf.

[9] Alumran A, Hurst C, Hou X-Y (2011). Antibiotics overuse in children with upper respiratory tract infections in Saudi Arabia: risk factors and potential interventions. Clin Med Diagn.

[10] el-Gilany AH (2000). Acute respiratory infections in primary health care centres in northern Saudi Arabia. East Mediterr Health J.

[11] Olwi RI, Olwi DI (2021). Trends in the use of antibiotics for pharyngitis in Saudi Arabia. J Infect Dev Ctries.

[12] Al-Niemat SI, Aljbouri TM, Goussous LS, Efaishat RA, Salah RK (2014). Antibiotic prescribing patterns in outpatient emergency clinics at Queen Rania Al Abdullah II children’s hospital, Jordan, 2013. Oman Med J.

[13] Amin MT, Abd El Aty MA, Ahmed SM, Elsedfy GO, Hassanin ES, El-Gazzar AF (2022). Over prescription of antibiotics in children with acute upper respiratory tract infections: a study on the knowledge, attitude and practices of non-specialized physicians in Egypt. PLoS One.

[14] Li J, Song X, Yang T, Chen Y, Gong Y, Yin X, Lu Z (2016). A systematic review of antibiotic prescription associated with upper respiratory tract infections in China. Medicine (Baltimore).

[15] Nowakowska M, van Staa T, Mölter A (2019). Antibiotic choice in UK general practice: rates and drivers of potentially inappropriate antibiotic prescribing. J Antimicrob Chemother.

[16] Rún Sigurðardóttir N, Nielsen AB, Munck A, Bjerrum L (2015). Appropriateness of antibiotic prescribing for upper respiratory tract infections in general practice: comparison between Denmark and Iceland. Scand J Prim Health Care.

[17] Al-Homaidan HT, Barrimah IE (2018). Physicians’ knowledge, expectations, and practice regarding antibiotic use in primary health care. Int J Health Sci (Qassim).

[18] Tham DW, Abubakar U, Tangiisuran B (2020). A systematic review of antibiotic prescription associated with upper respiratory tract infections in China. Eur J Pediatr.

[19] Adriaenssens N, Coenen S, Tonkin-Crine S, Verheij TJ, Little P, Goossens H (2011). European Surveillance of Antimicrobial Consumption (ESAC): disease-specific quality indicators for outpatient antibiotic prescribing. BMJ Qual Saf.

[20] Grimshaw JM, Thomas RE, MacLennan G (2004). Effectiveness and efficiency of guideline dissemination and implementation strategies. Health Technol Assess.

[21] Grol R, Grimshaw J (2003). From best evidence to best practice: effective implementation of change in patients’ care. Lancet.

[22] Al-Shawi MM, Darwish MA, Abdel Wahab MM, Al-Shamlan NA (2018). Misconceptions of parents about antibiotic use in upper respiratory tract infections: a survey in primary schools of the Eastern Province, KSA. J Family Community Med.

[23] Md Rezal RS, Hassali MA, Alrasheedy AA, Saleem F, Md Yusof FA, Godman B (2015). Physicians' knowledge, perceptions and behaviour towards antibiotic prescribing: a systematic review of the literature. Expert Rev Anti Infect Ther.

[24] van der Velden AW, Pijpers EJ, Kuyvenhoven MM, Tonkin-Crine SK, Little P, Verheij TJ (2012). Effectiveness of physician-targeted interventions to improve antibiotic use for respiratory tract infections. Br J Gen Pract.

[25] Thoolen B, de Ridder D, van Lensvelt-Mulders G (2012). Patient-oriented interventions to improve antibiotic prescribing practices in respiratory tract infections: a meta-analysis. Health Psychol Rev.

[26] Sadeq AA, Hasan SS, AbouKhater N (2022). Exploring antimicrobial stewardship influential interventions on improving antibiotic utilization in outpatient and inpatient settings: a systematic review and meta-analysis. Antibiotics (Basel).

[27] Al-Tawfiq JA, Alawami AH (2017). A multifaceted approach to decrease inappropriate antibiotic use in a pediatric outpatient clinic. Ann Thorac Med.

